# Novel Anthropometric Indices as Screening Tools for Obesity: A Study on Healthy Iranians

**DOI:** 10.1155/2023/6612411

**Published:** 2023-10-03

**Authors:** Toktam Alirezaei, Hamid Soori, Rana Irilouzadian, Hadis Najafimehr

**Affiliations:** ^1^Men's Health and Reproductive Health Research Center, Shahid Beheshti University of Medical Sciences, Tehran, Iran; ^2^Safety Promotion and Injury Prevention of Research Center, Cohort Health Employees Center, Shahid Beheshti University of Medical Sciences, Tehran, Iran; ^3^Burn Research Center, Iran University of Medical Sciences, Tehran, Iran; ^4^School of Medicine, Shahid Beheshti University of Medical Sciences, Tehran, Iran

## Abstract

**Background and Aims:**

Upper body fat distribution is more related to cardiometabolic diseases than central obesity. Neck circumference (NC) and neck-to-height ratio (NHtR) are two indicators of upper body obesity that are affordable, easy to obtain, highly reproducible, and more practical in the crowded health centers than the classic anthropometric indices.

**Methods:**

18–65-year-old individuals with no past medical history were included. After obtaining written informed consent, they were screened for hypertension, high blood glucose, and other abnormal laboratory results. Data were analyzed using SPSS and Mann–Whitney *U* test, Chi square test, Spearman's correlation coefficient, and ROC curve.

**Results:**

In our 2,812 participants, NC had the lowest area under the curve (AUC) in both male and female obese and overweight subjects. NHtR and hip circumference (HC) had the highest AUC in men and women with obesity, respectively. The highest sensitivity for overweight men and women belonged to waist circumference (WC) and waist-to-height ratio (WHtR), respectively, and for both males and females with obesity, NHtR had the highest sensitivity. The cutoff point of NHtR had the same value for males and females. HC and NHtR had the highest positive likelihood ratio (PLR) for obesity in men. In addition, HC and WC had the highest PLR for obesity in women.

**Conclusion:**

In this study, we revealed that NC had the lowest and NHtR and HC had the highest predictive value for obesity. Furthermore, for both males and females with obesity, NHtR had the highest sensitivity. HC had the highest PLR for obesity in both genders. Our results warrant prospective studies to evaluate the role of NHtR and other novel anthropometric indices in the risk of cardiometabolic diseases.

## 1. Introduction

Obesity, recently labelled as a pandemic, is associated with various health problems including cardiovascular diseases, musculoskeletal disorders, depression, and adverse outcomes [1, 2]. Obesity and overweight have increased in recent years in Iran. According to a meta-analysis conducted by Okati-Aliabad et al., the prevalence of obesity has increased from 17.74 in the time frame of 2007–2013 to 25.98 in the time frame of 2014–2020. The statistics for overweight prevalence is from 27.02 in 2007–2013 to 38.29 in 2014–2020. However, Iran was not among the top-three countries with the highest prevalence of obesity or overweight in the Middle East region in the time frame of 2014–2020 [3, 4].

Body mass index (BMI) is a measure used commonly for indicating nutritional status and obesity [5]. Recent studies suggested that BMI—which only depends on height and weight—cannot indicate body fat content accurately [6]. Anthropometric indices that evaluate central adiposity such as waist circumference (WC) and waist-to-height ratio (WHtR) have been suggested to correlate with cardiometabolic disorders more than BMI [7–10]. WC is associated with cardiovascular diseases and metabolic syndrome. However, this parameter cannot be measured in clinics because it is affected by the time that an individual has eaten or defecated and of respiration.

Fat distribution in the upper body is more strongly associated with cardiovascular diseases than visceral adipose tissue and central obesity [11]. This association may be the result of more free fatty acid release in the upper body compared to the abdomen [12, 13]. Abundant fat tissue in upper body results in oxidative stress and endothelial injury, and, as a result, chronic vascular disease [14]. The association of the upper body obesity with insulin resistance, hypertriglyceridemia, gout, and renal stones is well-established [15–17].

NC, as an indicator of upper body adiposity, is associated with systolic and diastolic blood pressure, total cholesterol, low-density lipoprotein (LDL), triglycerides, fasting blood glucose, uric acid levels, insulin resistance, metabolic syndrome, and nonalcoholic fatty liver disease (NAFLD), and, as a result, cardiovascular diseases [16, 18–20]. There is some controversy over the indication of using NC and its predictive role for body composition [21, 22]. NC is simple, cheap, fast to measure, highly reproducible, and less-varied in age compared to WC [23, 24]. It is more feasible in overcrowded clinics and more culturally accepted. Neck-to-height ratio (NHtR) is a novel anthropometric index that not only measures upper body adiposity but also adjusts the NC for height. NHtR has been shown to be more associated with liver fibrosis compared to NC [25].

Abdominal volume index (AVI) is another novel anthropometric index and indicator of central obesity that is shown to be correlated with diabetes mellitus and metabolic abnormalities [26–28]. AVI has been suggested as a screening tool for metabolic syndrome of adulthood in different populations [29, 30].

The relation between NHtR and other well-known anthropometric indices has not been previously evaluated in healthy population as far as we know. Moreover, the cutoff and correlation of anthropometric indices are highly related to ethnicity; so, we aim to determine the cutoff values of these novel anthropometric indices and to evaluate their predictive value for overweight and obesity which is defined by BMI among Iranian healthy population. We hypothesized that NHtR could be a better and more feasible index for evaluating obesity and overweight.

## 2. Methods

### 2.1. Participants

This is a cross-sectional study in which 3452 volunteer staff in Shohada-e Tajrish Hospital in Tehran, Iran, participated from 2021 to 2022. All participants were provided and signed the written informed consent. The study was approved by the Ethics Committee of the Shahid Beheshti University of Medical Sciences (Ethics code: IR.SBMU.MSP.RETECH.1400.721).

**Figure 1:**
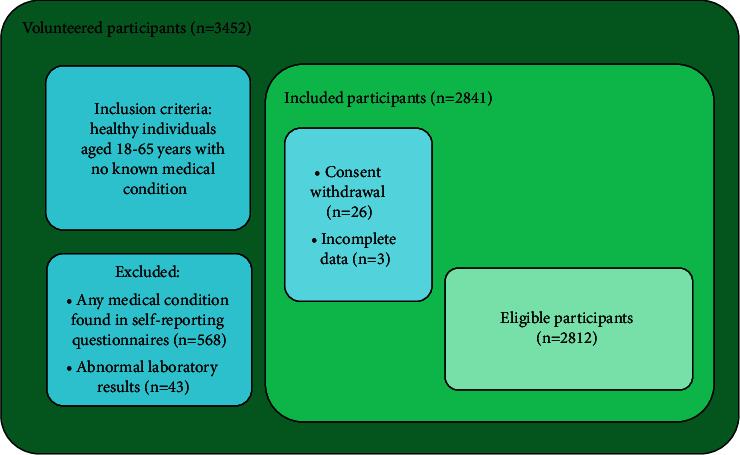
Flow chart of participants' selection.

All data were collected from individuals by interview, physical examination, and laboratory findings. Past medical history was obtained by a questionnaire, a blood sample in fasting state was taken, and anthropometric parameters were measured. Included individuals were 18–65 years old, volunteered, and with no known medical condition. Exclusion criteria included individuals with hypertension, diabetes mellitus, cancer, stroke, and hepatic, renal, cardiac, respiratory, and thyroid diseases, head and neck surgery and radiation, hematological disorders, abdominal diseases that might affect the distribution of fat, history of or current use of corticosteroids, Cushing's syndrome, and other disorders of the pituitary or adrenal glands. Finally, 2,812 subjects were eligible and enrolled in this study.  Figure 1 demonstrates the flow chart of participants' selection. The study was explained to participants and an informed written consent was obtained. The confidentiality of all of the collected data was respected. Participants were grouped by their BMI (kg/m^2^) into underweight (BMI < 18.5), normal-weight (18.5 ≤ BMI < 24.9), overweight (25 ≤ BMI < 29.9), and obese (BMI ≥ 30).

### 2.2. Data Collection

A blood sample was drawn from participants after at least 12 hours of fasting and was measured within hours of collection. The measured parameters were fasting blood sugar (FBS), complete blood cell count (CBC), lipid profile including low-density lipoprotein cholesterol (LDL), high-density lipoprotein cholesterol (HDL) and triglycerides (TG), creatinine (Cr), blood urea nitrogen (BUN), and uric acid (UA). InBody 770 analyzer was used to measure anthropometric parameters including weight, height, hip circumference (HC), WC, waist-to-height ratio (WHtR), NC, and NHtR. BMI was calculated by dividing weight (kg) to height squared (m^2^). Abdominal volume index (AVI) was computed using the following formula [31]:

(1) $$\begin{array}{c} \text{AVI}=\frac{\left[2\text{ cm}{\left(\text{WC}\right)}^{2}+0.7\text{ cm}{\left(\text{WC}-\text{HC}\right)}^{2}\right]}{1000}. \end{array}$$

### 2.3. Statistical Analysis

All statistical analyses were performed in SPSS software (version 23). The significance level was considered at *α* = 0.05 for the whole analysis. The descriptive statistics of each variable with normal and nonnormal distribution were presented in the form of the mean ± standard deviation (SD) and median (interquartile range (IQR)), respectively. The number and percentage of cases have also been reported. After checking the normality assumption, nonparametric tests were used for variables with nonnormal distribution. Average significant differences between men and women were checked by using the Mann–Whitney *U* test. The chi-square test was used to examine the relationship between BMI levels and sex. The multiple linear regression and Spearman's correlation coefficient (*r*) were used to examine the relationship between BMI and other anthropometric indicators. In addition, the receiver operating characteristic (ROC) curve was applied for checking the predictive validity and determination of cutoff points of each anthropometric indicator for identifying the subjects with overweight and obesity. All characteristics of ROC curve analysis (including area under the curve (AUC), 95% confidence interval (CI), sensitivity, and specificity) were performed. Some indexes such as the positive predictive value (PPV), positive likelihood ratio (LP), negative predicted value (NPV), and negative likelihood ratio (LN) for each indicator were also computed.

## 3. Results

A total of 2,812 subjects (male = 1057 (37.6%) and female = 1755 (62.4%)) were included in this study with the median age of 42 (IQR = 12.00) years. The median BMI for all participants was 26.68 (IQR = 5.5) which fell in the overweight category. BMI was not significantly different between male and female groups. Evaluation of BMI categories showed that nearly half of the participants (45.1%) were in the overweight category and the others were in order, in the normal (31.4%), obese (22.8%), and underweight (0.7%) categories. Other demographic details classified by gender can be seen in Table 1. The results of anthropometric indices did not show any difference between the two genders. Results of the correlation analysis, examining the relationship between BMI and other obesity indicators, are presented in Table 1. In each gender and the overall sample, significant positive correlations (*p* < 0.05) between BMI and all other anthropometric indices were found. In our study group, the highest positive correlation was observed between BMI and WHtR (*r* = 0.87), followed by HC (*r* = 0.86), AVI (*r* = 0.85), WC (*r* = 0.84), and NHtR (*r* = 0.68) (Table 1). In addition, the mean, median, and IQR of anthropometric indices in underweight, normal-weight, overweight, and obese groups are indicated in Table 2.

Regression analysis also revealed that about 91.0% of variations in BMI are explained by the anthropometric variables along with the TG and gender (Table 3). According to Table 3, AVI, HC, NC, and NHtR are independent predictors of BMI (all *p* values <0.05); each unit increase in NHtR results in 3.2 units increase in BMI.

**Figure 2:**
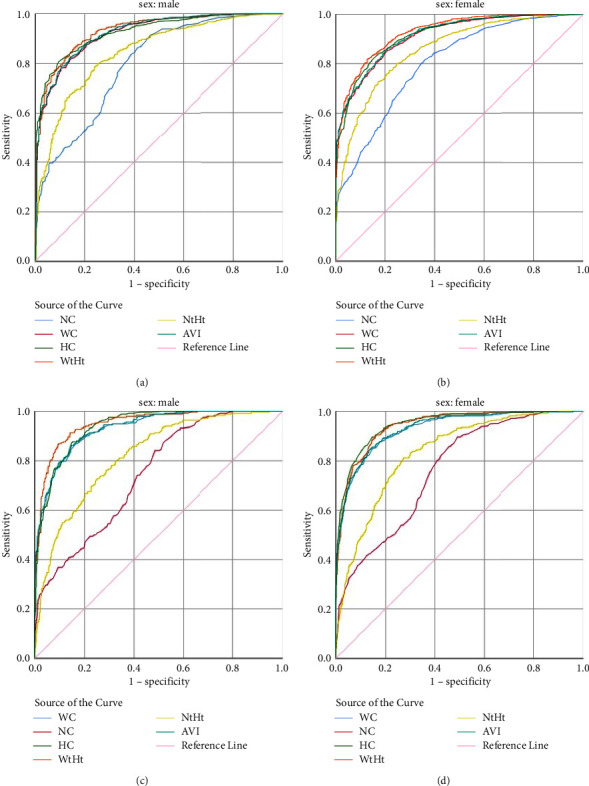
Receiver operating characteristic (ROC) curves of anthropometric parameters as an indicator of overweight ((a) male; (b) female) and obesity ((c) male; (d) female).

Figure 2 illustrates the accuracy of individual indicators in identifying overweight and obesity subjects by ROC curves, and Table 4 presents the information about the AUC of the curves. AUC results being more than 0.74 in both genders revealed that all of the indices had a high accuracy for screening individuals with overweight and obesity. The minimum AUC belonged to NC for overweight with AUC = 0.79 (SE = 0.015, 95% CI: 0.77–0.82) in men and with AUC = 0.80 (SE = 0.011, 95% CI: 0.78–0.82) in women and for obesity with AUC = 0.74 (SE = 0.020, 95% CI: 0.71–0.77) in men and with AUC = 0.76 (SE = 0.013, 95% CI: 0.74–0.78) in women. NHtR had the highest AUC in men with obesity with AUC = 0.95 (SE = 0.014, 95% CI: 0.94–0.96) and HC had the highest AUC in women with obesity with AUC = 0.95 (SE = 0.006, 95% CI: 0.93–0.96). WHtR, HC, and AVI same as WC had the highest predictive abilities in identifying overweight groups in order. For obesity, the highest predictive values are NHtR, HC, and WC same as AVI in men and HC, WHtR, and WC same as AVI in women (Table 4).

For overweight and obese groups, cutoff points, sensitivity, specificity, PPV, NPV, LP, and LN were determined. For overweight individuals, the cutoff points for NC, WC, HC, and AVI were higher for women (NC = 34.56, WC = 88.15, HC = 99.35, and AVI = 15.94) compared to men (NC = 33.85, WC = 85.15, HC = 98.26, and AVI = 15.56). For the cutoff points of NHtR and WHtR, the male and female overweight groups were similar. Sensitivity of WC and WHtR was the highest for overweight men and women, respectively.

For the individuals with obesity, the cutoff points for indices of WC, HC, WHtR, and AVI were larger for men (WC = 96.35, HC = 104.95, WHtR = 0.58, and AVI = 18.39) than women (WC = 95.95, HC = 104.55, WHtR = 0.56, and AVI = 18.18). NC and NHtR had the same cutoff for women and men with obesity (NC = 36.95 and NHtR = 0.22). Sensitivity of NHtR was the highest compared to other indices for both males and females with obesity.

In Table 5, the likelihood ratios for each cutoff point are also shown. For instance, a man with NC of more than 33.85 centimeters is 2.22 times more likely to be overweight than a man with NC value below this cutoff point.

HC and NHtR had the highest positive likelihood ratio for obesity in men (4.74 and 4.65, respectively). The highest positive likelihood ratio for women with obesity belonged to HC and WC, being 4.89 and 4.45, respectively.

## 4. Discussion

Obesity is a medical condition which is a risk factor for metabolic and cardiovascular diseases as well as adverse events and complications. In our study, 2,812 healthy subjects, 1,755 (62.4%) females, and 1,057 (37.6%) males were enrolled. Nearly half of the subjects were classified in overweight group (45.1%) and then, in order, normal weight (31.4%), obese (22.8%), and underweight (0.7%). Our results are in accordance with the prevalence reported in the meta-analysis in the study by Okati-Aliabad et al. ; the prevalence of overweight and obesity in recent years was reported to be 38.29 and 25.98, respectively [3].

In a study in healthy individuals with normal WC, significant variation in visceral adipose tissue was found and it was suggested that WC may not be a good indicator of the total body fat [32]. In a meta-analysis by Ashwell et al. in adults, they showed that WHtR is a better predictor than WC and BMI in terms of metabolic risk factors [33]. However, another meta-analysis among pediatric population showed that WHtR did not have any superiority to BMI and WC for predicting cardiometabolic disorders [34]. These findings suggested that novel anthropometric indices such as NHtR and AVI may have a better predictive value for metabolic syndrome and cardiovascular risk factors.

Recent studies revealed that upper body adipose tissue is more attributable to metabolic syndrome because increased free fatty acids in blood, which are a risk factor for cardiometabolic diseases, is mostly the result of the upper body subcutaneous fat lipolysis [35–37]. In addition, studies showed that upper body adipose tissue is related to endothelium dysfunction and vascular injury [14]. NC is a good index for measuring upper body adipose tissue and is not affected by the state of being eaten, defecation, respiratory and gastrointestinal disorders, and abdominal surgeries [38]. In a study by Preis et al. using the results of Framingham research, they have shown that NC, independent of WC and BMI, is associated with higher blood glucose, distorted lipid profile, high blood pressure, and cardiovascular diseases [39]. In a study among healthy Chinese adults, NC was found to be associated with insulin resistance and cardiometabolic risk factors [40]. Similar associations were found in other studies among different populations [41–43]. In a study by Mirr et al. among Caucasian population, indices of TyG-NC and TyG-NHtR were associated with metabolic syndrome [44]. We also performed a multivariate regression using TG, NC, NHtR, WC, HC, age, and gender which yielded significant correlation with BMI.

NHtR is an anthropometric index that uses NC and adjusts it for height. In a study by Mondal et al., NHtR was found to be superior to NC in predicting liver fibrosis, NAFLD, and metabolic syndrome [25]. In a study among patients with suspected cerebrovascular accident who had underwent computerized tomography angiography, NHtR was associated with mortality [45]. In another study, authors claimed that NC and NHtR are predictors of metabolic syndrome and its risk factors among Asian Indians [46].

In our study group, the highest positive correlation was observed between BMI and WHtR, followed by HC, AVI, WC, and NHtR. Moreover, AVI, HC, NC, and NHtR were independent predictors of BMI. Similarly, in a systematic review by Tellez et al., there is a positive correlation between NC and other obesity indices indirectly measured by CT scan and body analyzer devices in adults; however, they suggested that there is no indication for using NC in adolescents for predicting body composition [22]. In another study conducted by Khosravian et al., they showed that all anthropometric indices except NC had a significant difference between people with metabolic syndrome and those without metabolic syndrome. They also showed that WHtR had the highest AUC, followed by BMI, AVI, and WC for predicting metabolic syndrome [47].

We demonstrated that NHtR had a higher AUC compared to NC for predicting both overweight and obesity. Moreover, the minimum AUC in the ROC belonged to NC for male and female overweight and obese groups. NHtR had the highest AUC in men with obesity (AUC = 0.95); however, WHtR and HC had more predictive abilities in other groups.

In a study among 1,912 Turkish individuals, the NC cutoff values suggested for obesity were 34.5 cm for women and 38.5 cm for men [48]. In our study, the cutoff for obesity was 36.95 cm for both sexes which may suggest the differences attributable to ethnicity. Moreover, our results revealed that NC and NHtR had the same cutoff for women and men with obesity (NC = 36.95 and NHtR = 0.22). In male and female overweight groups, the cutoff points of NHtR and WHtR were similar.

In men and women with obesity, NHtR of 0.22 had the highest sensitivity (93% and 95%, respectively) with an acceptable specificity (80% and 70%, respectively) compared to other indices. In overweight men and women, the highest sensitivity belonged to WC and WHtR, respectively.

HC and NHtR had the highest positive likelihood ratios for obesity in men (4.74 and 4.65, respectively). The highest positive likelihood ratios for women with obesity belonged to HC and WC, being 4.89 and 4.45, respectively.

Our study revealed that AVI had the same AUC as WC, both lesser than WHtR and HC, in both male and female obese and overweight groups. Studies revealed that AVI is associated with impaired glucose metabolism [28]. A study by Wu et al. [49] demonstrated that AVI had the highest predictive value for metabolic syndrome and low HDL in nonoverweight/obese male (AUC = 0.743) and female (AUC = 0.819) subjects. They also proposed that AVI was the best predictor of insulin resistance impaired glucose tolerance. For predicting metabolic syndrome in a similar study by Duan et al., AVI had the AUC of 0.838 and 0.794 for men and women, respectively [50]. In a study by Hu et al., AVI, as an index for abdominal adiposity, was associated with diabetes mellitus [29]. In a study by Hajian-Tilaki et al., they demonstrated that AVI and WC had the same mean between males and females, while the means of BMI and WHtR were different between the two groups. WHtR in women and AVI, WC, and WHtR in men were the predictors of 10-year cardiovascular disease risk, but not BMI [51]. Considering these results and the fact that obtaining WHtR and NHtR is easier than calculating AVI and BMI, they can be used by physicians during physical examination as a screening tool for cardiometabolic disorders.

Our study has some limitations. First, this is a cross-sectional study among healthy Iranian population. Therefore, it has the limitations of an observational study. Second, the results of this study may not be attributable to other ethnicities. Also, we did not follow-up the participants in the next years in order to evaluate metabolic syndrome incidence; this may propose future cohort studies to follow-up healthy individuals for assessing incidence of cardiometabolic disorders. Moreover, the results of our study suggest further investigations to evaluate NHtR as a diagnostic criterion of metabolic syndrome and a risk factor for ischemic heart diseases.

## Figures and Tables

**Table 1:** Main characteristics of participants by sex and correlation between anthropometric parameters and BMI.

	Total (*n* = 2812)	Male (*n* = 1057)	Female (*n* = 1755)	*p* value	Correlation with BMI		
					Male	Female	Total
Age, years, median (IQR)	42 (12.00)	42 (13.00)	41 (13.00)	0.037	0.03	−0.03	−0.01
TG, median (IQR)	109 (86)	112 (86)	107 (86)	0.336	0.29^*∗*^	0.27^*∗*^	0.28^*∗*^
Height, cm, median (IQR)	163 (12.90)	163 (13.50)	162.10 (12.80)	0.046	—	—	—
Weight, kg, median (IQR)	71.90 (19.10)	72.50 (19.60)	71.40 (18.80)	0.105	—	—	—
BMI, kg/m^2^, median (IQR)	26.68 (5.51)	26.83 (5.70)	26.56 (5.38)	0.175	—	—	—
Underweight, *n* (%)	19 (0.7%)	5 (26.3)	14 (73.7)	0.44	—	—	—
Normal, *n* (%)	881 (31.4%)	320 (36.3)	561 (63.7)		—	—	—
Overweight, *n* (%)	1269 (45.1%)	479 (37.7)	790 (62.3)		—	—	—
Obese, *n* (%)	640 (22.8%)	253 (39.5)	387 (60.5)		—	—	—
NC, cm, median (IQR)	35.50 (6.00)	35.70 (6.35)	35.30 (5.80)	0.161	0.57^*∗*^	0.55^*∗*^	0.56^*∗*^
WC, cm, median (IQR)	92 (15.50)	92.30 (15.70)	91.80 (15)	0.102	0.86^*∗*^	0.83^*∗*^	0.84^*∗*^
HC, cm, median (IQR)	102 (9.90)	102 (10.45)	102 (9.70)	0.707	0.86^*∗*^	0.85^*∗*^	0.86^*∗*^
NHtR, median (IQR)	0.22 (0.03)	0.22 (0.03)	0.22 (0.03)	0.122	0.68^*∗*^	0.67^*∗*^	0.68^*∗*^
WHtR, median (IQR)	0.56 (0.08)	0.56 (0.08)	0.56 (0.08)	0.214	0.87^*∗*^	0.86^*∗*^	0.87^*∗*^
AVI, median (IQR)	17 (5.60)	17.13 (5.74)	16.94 (5.41)	0.106	0.86^*∗*^	0.83^*∗*^	0.85^*∗*^

^*∗*^Significant. AVI, abdominal volume index; BMI, body mass index; HC, hip circumference; IQR, interquartile range; NC, neck circumference; NHtR, neck-to-height ratio; TG, triglycerides; WC, waist circumference; WHtR, waist-to-hip ratio.

**Table 2:** Measures of central tendency of anthropometric indices.

Indices	Underweight	Normal	Overweight	Obese
BMI; mean/median (IQR)	17.90/18.12 (0.87)	22.73/23.02 (2.33)	27.29/27.16 (2.43)	33.41/32.70 (3.69)
AVI; mean/median (IQR)	10.54/10.39 (2.14)	13.78/13.61 (2.82)	17.52/17.41 (3.41)	22.65/22.23 (4.12)
HC; mean/median (IQR)	88.25/88.50 (6.40)	95.57/95.80 (6.45)	102.74/102.50 (5.90)	112.36/112 (7.50)
NC; mean/median (IQR)	31.31/31.10 (2.80)	33.46/32.80 (3.80)	36.53/36.00 (5.90)	38.88/38.30 (6.35)
NHtR; mean/median (IQR)	0.18/0.19 (0.02)	0.20/0.20 (0.02)	0.22/0.22 (0.02)	0.24/0.23 (0.02)
WHtR; mean/median (IQR)	0.43/0.44 (0.05)	0.50/0.50 (0.05)	0.56/0.57 (0.05)	0.65/0.63 (0.07)

AVI, abdominal volume index; BMI, body mass index; HC, hip circumference; IQR, interquartile range; NC, neck circumference; NHtR, neck-to-height ratio; TG, triglycerides; WC, waist circumference; WHtR, waist-to-hip ratio.

**Table 3:** Multivariate regression analysis for the prediction of BMI using anthropometric and demographic variables.

Variable	Model 1			Model 2		
	Beta (95% CI)	*p* value	Adjusted *R*^2^	Beta (95% CI)	*p* value	Adjusted *R*^2^
Gender	—			0.13 (0.007–0.24)	0.039	0.915
TG	—			0.01 (0–0.002)	0.001	
NC	−0.48 (−0.77–0.18)	0.001	0.913	−0.45 (−0.78–−0.11)	0.010	
WC	−0.08 (−0.21–0.04)	0.205		−0.09 (−0.24–0.06)	0.237	
HC	0.30 (0.29–0.31)	<0.001		0.30 (0.29–0.31)	<0.001	
NHtR	2.68 (1.66–7.70)	<0.001		3.20 (2.69–4.70)	<0.001	
WHtR	3.85 (−14.73–22.44)	0.684		6.13 (−15.43–27.70)	0.577	
AVI	0.53 (0.40–0.65)	<0.001		0.49 (0.35–0.63)	<0.001	

Model 1 is a regression model including just anthropometric indicators (TG, NC, WC, HC, NHtR, WHtR, and AVI); model 2 adds TG and gender to the predictors of model 1. AVI, abdominal volume index; BMI, body mass index; HC, hip circumference; NC, neck circumference; NHtR, neck-to-height ratio; TG, triglycerides; WC, waist circumference; WHtR, waist-to-hip ratio.

**Table 4:** Areas under the curve (AUC) for identifying subjects with overweight and obesity based on different anthropometric indicators by sex.

Anthropometric indicators	Male				Female			
	AUC	SE	95% CI	*p* value^*∗*^	AUC	SE	95% CI	*p* value^*∗*^
Overweight								
NC	0.79	0.015	0.77–0.82	<0.001	0.80	0.011	0.78–0.82	<0.001
WC	0.92	0.009	0.90–0.93	<0.001	0.91	0.007	0.90–0.92	<0.001
HC	0.93	0.008	0.91–0.94	<0.001	0.92	0.007	0.90–0.93	<0.001
WHtR	0.94	0.008	0.92–0.95	<0.001	0.93	0.006	0.91–0.94	<0.001
NHtR	0.84	0.013	0.82–0.87	<0.001	0.86	0.009	0.84–0.87	<0.001
AVI	0.92	0.008	0.91–0.94	<0.001	0.91	0.007	0.90–0.93	<0.001
Obese								
NC	0.74	0.020	0.71–0.77	<0.001	0.76	0.013	0.74–0.78	<0.001
WC	0.93	0.008	0.91–0.95	<0.001	0.93	0.007	0.91–0.94	<0.001
HC	0.94	0.007	0.92–0.95	<0.001	0.95	0.006	0.93–0.96	<0.001
WHtR	0.82	0.007	0.79–0.85	<0.001	0.94	0.006	0.93–0.95	<0.001
NHtR	0.95	0.014	0.94–0.96	<0.001	0.83	0.011	0.82–0.95	<0.001
AVI	0.93	0.008	0.92–0.95	<0.001	0.93	0.007	0.92–0.95	<0.001

^*∗*^Null hypothesis: true area = 0.5. AUC, area under the curve; AVI, abdominal volume index; BMI, body mass index; HC, hip circumference; NC, neck circumference; NHtR, neck-to-height ratio; WC, waist circumference; WHtR, waist-to-hip ratio.

**Table 5:** Optimal cut-off points, sensitivity, and specificity of different anthropometric indicators for identifying overweight and obesity in male and female patients.

Anthropometric indicators	Cutoff point	Sensitivity (%)	1-Specificity (%)	PPV (%)	NPV (%)	LP	LN
Overweight males							
NC	33.85	80	36	56	89	2.22	0.31
WC	85.15	92	30	57	90	3.07	0.11
HC	98.25	90	26	65	88	3.46	0.14
WHtR	0.53	90	22	59	83	4.09	0.13
NHtR	0.21	80	26	70	89	3.08	0.27
AVI	15.56	86	19	72	90	4.53	0.17
Obese males							
NC	36.95	60	35	56	83	1.71	0.62
WC	96.35	90	20	57	89	4.50	0.13
HC	104.95	90	19	61	91	4.74	0.12
WHtR	0.58	76	30	55	87	2.53	0.34
NHtR	0.22	93	20	54	92	4.65	0.09
AVI	18.39	90	25	59	89	3.60	0.13
Overweight females							
NC	34.56	70	27	60	80	2.59	0.41
WC	88.15	83	19	55	83	4.37	0.21
HC	99.35	85	20	57	81	4.25	0.19
WHtR	0.53	86	19	61	89	4.53	0.17
NHtR	0.21	80	26	70	91	3.08	0.27
AVI	15.94	81	16	72	90	5.06	0.23
Obese females							
NC	36.95	61	23	55	90	2.65	0.51
WC	95.95	89	20	60	92	4.45	0.14
HC	104.55	93	19	59	86	4.89	0.09
WHtR	0.56	80	26	66	80	3.08	0.27
NHtR	0.22	96	30	64	83	3.20	0.06
AVI	18.18	90	21	60	92	4.29	0.13

AVI, abdominal volume index; BMI, body mass index; HC, hip circumference; LN, negative likelihood; LP, positive likelihood; NC, neck circumference; NHtR, neck-to-height ratio; NPV, negative predictive value; PPV, positive predictive value; WC, waist circumference; WHtR, waist-to-hip ratio.

## Data Availability

The data that support the findings of this study are available on request from the first author.

## Additional Points

*Research Highlights.* (i) Neck-to-height ratio and hip circumference had the highest predictive value for men and women with obesity, respectively. (ii) Neck circumference had the lowest predictive value for identifying overweight and obesity. (iii) Hip circumference had the highest positive likelihood ratio for obesity in men and women.
